# The evolving role and utility of off-label drug use in multiple myeloma

**DOI:** 10.37349/etat.2021.00050

**Published:** 2021-08-30

**Authors:** James H Stoeckle, Faith E Davies, Louis Williams, Eileen M Boyle, Gareth J Morgan

**Affiliations:** Perlmutter Cancer Center, New York University Langone Health, New York, NY 10016, USA; IRCCS National Cancer Institute (NCI), Italy

**Keywords:** Multiple myeloma, off-label, drug development, orphan drugs, thalidomide, relapsed refractory

## Abstract

The treatment landscape for multiple myeloma (MM) has dramatically changed over the last three decades, moving from no US Food and Drug Administration approvals and two active drug classes to over 19 drug approvals and at least eight different active classes. The advances seen in MM therapy have relied on both a structured approach to obtaining new labels and cautious off-label drug use. Although there are country and regional differences in drug approval processes, many of the basic principles behind off-label drug use in MM can be summarized into four main categories: 1) use of a therapy prior to the current approval regulations; 2) widespread use of a therapy following the release of promising clinical trial results but prior to drug approval; 3) use of a cheap therapy supported by clinical safety and efficacy data but without commercial backing; and 4) niche therapies for small well-defined patient populations where large clinical trials with sufficient power may be difficult to perform. This review takes a historical approach to discuss how off-label drug use has helped to shape the current treatment approach for MM.

## Introduction

There are currently 19 drugs approved by the US Food and Drug Administration (FDA) for the treatment of multiple myeloma (MM) [1] (Table 1). This portfolio represents a marked increase since the 1990s, when the only therapies available were alkylating agents—such as melphalan and cyclophosphamide—corticosteroids, and autologous stem cell transplantation (ASCT)–none of which had been formally reviewed by the FDA. Although the process for drug approval and widespread clinical use varies depending on geographical region, the basic principles behind requiring regulatory permission are now universal and important to ensure the safety and efficacy of new therapies.

**Table 1.** List of drugs used in MM with FDA approval and labeled indications

**Novel agents**	**Year approved**	**Indication (lines of therapy failed)**	Approved in combination*
Thalidomide	1998	NDMM	Dexamethasone
Bortezomib (IV and SC)	2003	NDMM RRMM	Melphalan/prednisone
Lendalidomide	2005	RRMM Maintenance	Dexamethasone
Carfilzomib	2012	RRMM (1–3)**	Lenalidomide/dexamethasone Daratumumab/dexamethasone Dexamethasone
Pomalidomide	2013	RRMM (2)	Dexamethasone
Panobinostat	2015	RRMM (2)	Bortezomib/dexamethasone
Daratumumab (IV and SC)	2015	NDMM	Lenalidomide/dexamethasone Bortezomib/melphalan/prednisone Bortezomib/melphalan/dexamethasone
		RRMM (1)	Lenalidomide/dexamethasone Bortezomib/dexamethasone
		RRMM (1–3)** RRMM (2)	Carfilzomib/dexamethasone Pomalidomide/dexamethasone
Elotuzumab	2015	RRMM	Lenalidomide/dexamethasone Pomalidomide/dexamethasone
Ixazomib	2015	RRMM	Lenalidomide/dexamethasone
Selinexor	2019	RRMM (1) RRMM (PR)	Bortezomib/dexamethasone Dexamethasone
Belantamabmafodotin	2020	RRMM (4)	
Isatuximab	2020	RRMM	Pomalidomide/dexamethasone

**Cytotoxic chemotherapy**			

Cyclophospha-mide (IV and PO)	1959	MM (unspecified)	
Melphalan (IV and PO)	1964	Palliative ASCT	
Carmustine	1977	Palliative	Prednisone
Liposomal doxorubicin	1995	RRMM (1)	Bortezomib

**Adjunctive therapy**			

Zoledronic acid	1964		
Pamidronate	1995		
Plerixafor	2008		

* If no drugs listed in table under Approved in combination then approval was for use as single agent;

** FDA approval includes use as single agent. PR: penta-refractory; IV: intravenous; SC: subcutaneous; PO: oral; NDMM: newly diagnosed multiple myeloma; RRMM: relapsed/refractory multiple myeloma. Check FDA website and company prescribing for up-to-date details

However, many therapeutic advances have not only relied on this structured approach to obtaining new approvals but have also utilized off-label drug use. For example, in the US, once approved by the FDA healthcare providers may prescribe a drug for an indication outside of those included in the FDA label when they judge that it is medically appropriate. The history of drug development in MM over the last 30 years provides a striking example of how the common prescribing practice of off-label drug use [2–5] is used within a single disease setting, how this practice impacts the introduction of novel therapies, results in varied clinical trial designs, and eventually modifies clinical practice.

**Figure 1.**
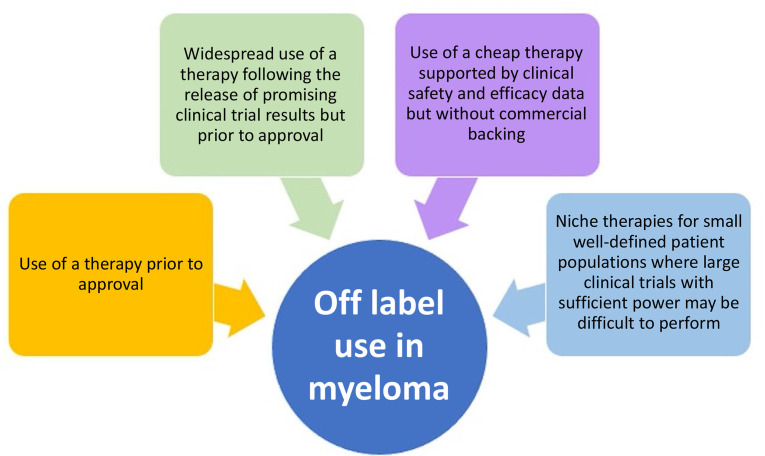
Reasons for off-label drug use in MM

The potential reasons for non-approved use can be summarized into four main categories: 1) use of a therapy prior to the current regulations; 2) widespread use of a therapy following the release of promising clinical trial results but prior to drug approval; 3) use of a cheap therapy supported by clinical safety and efficacy data but without commercial backing; and 4) niche therapies for small well-defined patient populations where large clinical trials with sufficient power may be difficult to perform (Figure 1). Regulatory approvals change frequently and as discussed above are country specific. This review will mainly discuss US FDA approvals, and although the specifics of a drug’s label and its off-label use may differ by region, the basic principles behind off-label drug use are universal.

## Drugs in regular use in MM without an FDA label

Prior to the current MM therapeutic landscape, where multiple FDA-approved treatment options are available, the predominant drugs in use were corticosteroids and alkylating agents for which there were no formal approvals. Following the introduction of low doses of the alkylating agent melphalan, high-dose melphalan (HDM) was developed [6]. The toxicity of HDM was subsequently modified by the use of stem cell rescue with ASCT which improved time to blood count recovery and improved rates of infection and mortality [7, 8]. Although the escalating doses of melphalan were associated with high toxicity, for the first time, therapy in MM was associated with deep responses and healing of bone lesions.

The widespread uptake of HDM with ASCT was based on the results of a series of three studies which randomized standard dose chemotherapy against ASCT [9–11]. These trials showed improved progression-free survival (PFS) compared with ASCT with a potential benefit for overall survival (OS). Importantly, HDM with ASCT changed the aim of therapy from the initial goal of disease control to one which recognized that deep responses translated into improved outcomes [12, 13]. The doses of the alkylator cyclophosphamide and its associated drug combinations used most commonly in clinical practice are not FDA-approved. Off-label use of cyclophosphamide in MM is broad and includes MM renal impairment or amyloid, or as a cheaper third drug in a triplet combination [14, 15].

Corticosteroids have served as a critical backbone in the treatment of MM, from the early era to the current day. Dexamethasone is active as a single agent [16] and as part of drug combinations. The FDA label has a nonspecific indication for use in combination with other myeloma-directed therapies [17]. A series of studies showed dexamethasone improved response rates that were not maintained long term [18, 19], but it remained a backbone drug because of the importance of response rates during the drug approval process. Prednisone, which lacks an FDA label for MM, was the corticosteroid of choice when used in combination with low dose melphalan in the combination melphalan-prednisone (MP) [20], whereas dexamethasone was preferentially used with vincristine and doxorubicin as vincristine-doxorubicin-dexamethasone (VAD) [21]. Maximizing the dexamethasone dose was associated with better responses and a dose of 40 mg daily on days 1–4, 9–12, and 17–20 became a standard in MM [22]. In this setting 70% of patients resistant to melphalan and 33% of those resistant to both melphalan and doxorubicin were noted to have a response. Later co-operative group studies of dexamethasone combinations with immunomodulatory drugs [23] (see below) highlighted some of the infections associated with dexamethasone. Refined dosing emerged, with once weekly dosing becoming more common (e.g., 40 mg) and a lower dose for older, less fit patients (e.g., 10–20 mg) to minimize infective and neurologic consequences.

The cytotoxic chemotherapeutics vincristine and doxorubicin used in VAD still lack an FDA label. Though the liposomal form of doxorubicin has been approved, it is no longer in widespread use in the clinic [24]. These agents have been successfully combined with novel agents to generate the Velcade-thalidomide-dexamethasone-cisplatin-doxorubicin-cyclophosphamide-etoposide (VTD-PACE) regimen (bortezomib, thalidomide, dexamethasone, cisplatin, doxorubicin, cyclophosphamide, etoposide). Like vincristine and doxorubicin, the cytotoxic agents etoposide, cisplatin, and cyclophosphamide used in the VTD-PACE regimen still do not have an FDA label for MM. Despite this they are widely used for patients with resistant and refractory disease where good responses are generated [25], and in the upfront setting as part of the total therapy regimens where excellent responses and clinical outcomes are seen [26].

## The novel drug era

**Figure 2.**
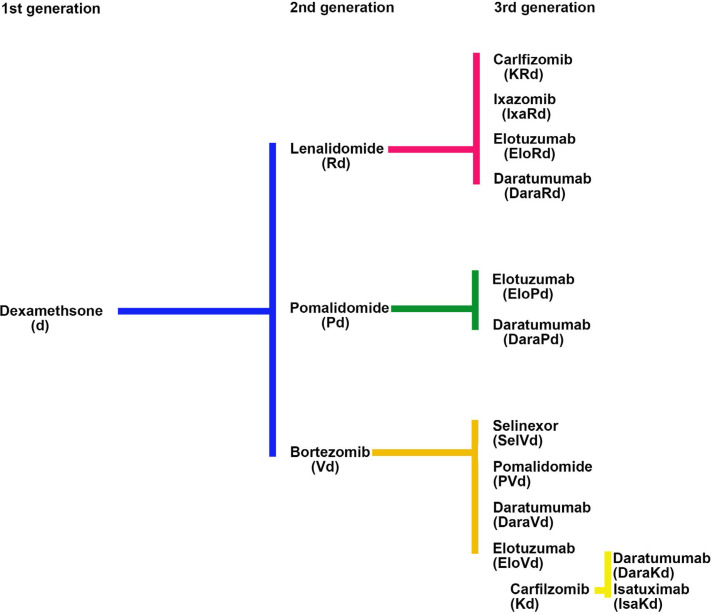
History of comparator arms in relapsed refractory trials (1–3 prior lines)

The development of the proteasome inhibitor (PI) bortezomib, and the immunomodulatory inhibitory drug (IMiD) lenalidomide in MM illustrates how new drugs were introduced into a setting where the primary therapies in use did not have an FDA label and how off-label drug use plays an important role in the drug development process. For both bortezomib and lenalidomide the initial challenge was to choose the most appropriate control drug for comparison with novel agents in the relapsed clinical setting where there was an unmet need for new therapies. Single agent dexamethasone, without an FDA label for this indication, was chosen over alkylating agents because of its ease of use and less myelosuppressive side effect profile (Figure 2).

### The immunomodulatory drugs

Thalidomide was developed in the mid-1950s for use as a sedative with minimal side effects or addictive potential [27]. By 1961 it was taken off the market worldwide after teratogenic effects were noted; close to 10,000 infants were thought to have limb deformities caused by the drug. The observation that thalidomide resulted in teratogenesis led investigators to consider its effect as an antineoplastic drug, though early efforts failed to show significant activity in advanced cancer [28, 29].

Thalidomide was initially successful as a treatment for erythema nodosum leprosum and inflammatory ulcers. Decreased tumor necrosis factor-alpha and inhibition of vascular endothelial growth factor and fibroblast growth factors were found to be the mechanism for anti-inflammatory and antiangiogenetic properties. In the 1990s, the antiangiogenic properties of thalidomide were discovered [30] and its promise as a cancer therapeutic emerged largely through kismet. A patient’s family member noticed the association of thalidomide activity with anti-angiogenesis and, also noted that MM was associated with an excess of neovascular formation. Following a conversation with their doctor a clinical trial in MM was initiated [31]. Later, it was shown that other mechanisms of action of thalidomide included impairment of cell-cell signaling, inhibition of cytokines important to plasma cell growth proliferation, free radical-mediated DNA damage, and enhancement of T and natural killer (NK) cell responses [32–35]. However, it was years from this time point until the mechanism of action of thalidomide was fully understood, when cereblon, a receptor of the E3 ubiquitin ligase complex, was identified as a thalidomide-binding protein leading to proteasomal degradation of key plasma cell transcription factors Ikaros and Aiolos [31, 36, 37].

Early clinical trials demonstrated responses with single agent thalidomide. Among 84 patients with refractory MM, thalidomide had an overall response rate (ORR) of 32% with two complete responses [38]. A number of studies compared thalidomide to dexamethasone and evaluated the two drugs in combination in the relapsed setting [39, 40]. As initial therapy, thalidomide in combination with doxorubicin and dexamethasone (TAD) was compared to what was then the European standard induction regimen for transplant eligible patients, VAD [41]. The superiority of TAD had a significant impact on the therapeutic landscape, ultimately leading to the “death of VAD” as a clinically used regimen, the licensing of thalidomide in combination with dexamethasone in 2006, and the development of the Risk Evaluation and Mitigation Strategies (REMS) program [42]. The REMS program is now in widespread use to prevent exposure of “at risk” individuals such as pregnant women to thalidomide, and the principle to decrease exposure has been extended to other anti-cancer drugs that may also be considered at high risk of teratogenicity.

Since the introduction of thalidomide and its derivative lenalidomide, IMiDs have become a critical backbone of standard induction regimens. In the process of introducing these drugs it was shown in relapsed patients that a lenalidomide-dexamethasone combination was more effective when compared to dexamethasone alone [43–45]. A third thalidomide derivative, pomalidomide, was developed in a similar fashion, leveraging off-label use of dexamethasone as its control arm, and has become a key component of treatment for relapsed disease.

Prior to the official approval of IMiDs, they were widely employed in combination with dexamethasone by investigators in clinical trials, leading to greater experience and optimization of their use. IMiDs were initially given in a continuous fashion as maintenance for patients post-HDM with ASCT, and following the combination of melphalan, thalidomide and prednisone for non-transplant candidates before formal approval in these settings. The side effect profile of thalidomide, however, was not ideal for long term maintenance therapy after investigator-initiated trials noted toxicities such as constipation, somnolence, and neuropathy [46] and it was soon replaced by lenalidomide. A number of clinical trials explored lenalidomide as maintenance following HDM and ASCT [47–49] using two different dosing regimens: 10–15 mg continuously and 10 mg for 21 out of 28 days. Its use was associated with myelosuppression that led to discontinuation as maintenance, especially in older patients [49]. Intermittent dosing was associated with less myelosuppression [48], and this became the regimen now widely in use for maintenance, despite lenalidomide not having an FDA label for this dose schedule [50].

The efficacy of combination therapy with IMiDs and dexamethasone in relapsed MM provided a new FDA-approved comparator group for future clinical trials, allowing the field to move from the use of doublet combinations to combinations based on two new agents together with dexamethasone. Each successive improvement was compared to the previous standard of care, all relating back to the initial comparison to dexamethasone used without an FDA label (Figure 2).

### The proteasome inhibitors

The development of PIs took a parallel and, in some ways, competitive path to the development of the IMiDs. The initial phase II trial that led to the approval of bortezomib [51] evaluated a dose of 1.3 mg/m^2^ on days 1, 4, 8 and 11 intravenously. This dosing schedule was associated with significant peripheral neuropathy [52] and a dose reduction was incorporated into future clinical trials to abrogate the toxicity. Despite the side effect profile, the original dose was approved [53], and was the dosing schedule chosen for the drug’s subsequent development, for example with melphalan as part of the Velcade-MP (VMP) regimen [54]. As it was the approved dosage all subsequent regulatory trials were compared to this dose and schedule even though clinicians learned to manage the toxicity by using off-label weekly dosing, and rapidly switched to the use of subcutaneous injections when a preparation was available. Therefore, outside of the regulatory setting the dose was reduced to 1.3 mg/m^2^ subcutaneously at weekly intervals–dosing that has never been formally tested against a control in a randomized trial and is based on small phase II datasets [55–57]. The use of standardized dosages during the approval process is important; however, this need has to be balanced against knowledge gained by real-world drug use obtained post-FDA approval to minimize side effects. In this case the newer dosing frequency decreased the rate of neuropathy and prolonged the length of time a patient was able to remain on drug. Such an approach does cause confusion when subsequent lines of therapy are developed, as ideally the original dosing schedule in the FDA label should be used as a standard against which to compare new therapies.

### Developing and utilizing the concept of RRMM

An important theme of drug development in MM has been the strategy of introducing successive waves of potentially useful clinical drugs for relapsed patients who have received all available therapies–so called “areas of unmet clinical need”. As discussed above, the therapies available 5–10 years ago included an IMiD, a PI and possibly an alkylator. In the early 2000s, once a drug was shown to be effective in patients with relapsed or refractory disease, combinations of the new drug with known effective drugs were developed including doublet, triplet and quadruplet combinations. These combinations were then examined in an earlier relapse setting before moving to the newly diagnosed setting.

By 2010–2015 with an increasing number of effective therapies, the need for a different approach with tighter definitions of eligible patients was needed, leading to the concept of relapsed/refractory MM (RRMM) in drug development. This was defined as progressive (within 60 days) or refractory disease during or after the receipt of prior therapy according to strict International Myeloma Working Group (IMWG) criteria [58–60]. Retrospective data analyses showed that as a group RRMM was associated with an extremely poor prognosis. In a cohort of 543 patients who had received a median of four prior lines of therapy, median PFS was 5 months and OS was 13 months [61]. This constituted a well-defined group with an unmet medical need that could provide a path forward for rapid new drug approval. After testing novel drugs alone and in combination in this setting, they could be then moved forward for evaluation in patients with RRMM after 1–3 prior lines of therapy, and subsequently for NDMM.

### Anti-CD38 antibodies

Anti-CD38 monoclonal antibodies have moved rapidly from an experimental drug to standard of care in various MM disease settings [62]. The initial studies of daratumumab showed a 30% ORR as a single agent in RRMM [63–65]. Since this observation, the drug has undergone extensive clinical trials in the relapsed setting (1–3 lines of therapy) where it has been combined with lenalidomide and dexamethasone (Rd) [66], bortezomib and dexamethasone (Vd) [67], carfilzomib and dexamethasone [68], as well as pomalidomide and dexamethasone [69, 70]. Isatuximab, another anti-CD38 antibody, is approved for use in combination with pomalidomide and dexamethasone [71] and is undergoing clinical trials with other drug combinations. Similar combinations have recently been approved in the upfront setting including daratumumab-Velcade-melphalan-prednisone (D-VMP), daratumumab-Revlimid-dexamethasone (DRd) for transplant ineligible patients, and daratumumab-Velcade-thalidomide-dexamethasone (D-VTD) for transplant eligible patients [72–74]. Compared to Europe where D-VMP, DRd and D-VTD are widely used for NDMM patients, in the US, Velcade-Revlimid-dexamethasone (VRD) is the standard of care for both patient groups. Although not yet FDA-approved, results from the Griffin study and extrapolation of other combination data showing good efficacy and safety profiles have led to the widespread use of D-VRD upfront [75]. Of note the FDA approvals for the subcutaneous form of daratumumab compared to the intravenous form also differ slightly [76, 77]. This is again due to the timing differences in the introduction of the two formulations. Even though the data concerning combinations with subcutaneous daratumumab upfront are awaited, there has been widespread uptake of this formulation in clinical practice, driven by the huge difference in infusion time (4–8 h *vs.* 5 min) and the advantages this has for both patients and clinics.

### Developing the concept of penta-refractory disease

Once anti-CD38 agents became widely available the clinical nature and history of patients with RRMM changed and the old definition of RRMM was no longer appropriate. This resulted in two new functional definitions, which are currently still in use: triple class-exposed (prior IMiD, PI, and anti-CD38 antibody) and penta-refractory (prior exposure and refractoriness to bortezomib, both lenalidomide and pomalidomide, and daratumumab). An updated analysis of RRMM outcomes following the introduction of daratumumab identified a median OS of only 5.6 months for patients considered as triple-exposed and penta-refractory [78]. Again, this represented a new area of unmet medical need where patients continue to have an extremely poor outcome after exposure and refractoriness to available therapies.

Clinical trials are difficult to perform in this area as patients tend to be unwell, and a full prior medical history with access to detailed medical records is required to ensure that patients meet the stringent definitions. It has also become clear that although this is a well-defined group, not all patients have the same biological disease. For example, patients may become penta-refractory over a 10-year period having received each drug sequentially or they may reach the same clinical state quickly over an 18-month period having received two different combinations of therapy. In addition, these definitions do not take into account clinical characteristics such as extramedullary disease or plasma cell leukemia, or genetic features such as mutations or translocations involving *MAF*, *NSD2*, *MYC*, or *TP53*.

Despite such difficulties, two drugs have been developed in this setting. The first is an antibody-drug conjugate belantamab mafadotin [79] that targets the B-cell maturation antigen (BCMA) which is highly expressed on MM cells [80, 81]. The anti-BCMA monoclonal antibody is conjugated to monomethyl auristatin, a microtubule disrupter resulting in targeted MM cell death. As a single agent in RRMM it showed a 30–34% ORR depending on the dose used [79]. The second therapy developed in this setting is the nuclear export inhibitor Selinexor [82], which blocks exportin 1, promoting apoptosis of malignant plasma cells by maintaining tumor suppressor proteins within the cell nucleus. In a trial of 122 patients with RRMM who were at least triple-class-exposed, selinexor combined with dexamethasone produced an ORR of 26% including two stringent complete responses. Given the activity of these therapies, use outside of their label will likely occur, i.e., in patients who fail to meet the strict criteria of penta-refractory disease but who in the physician’s eyes are likely to benefit from therapy.

### The challenge moving forward with new drug development

In addition to belantamab mafadotin and selinexor, a number of other drugs with novel mechanisms of action are also being developed in this penta-refractory setting, including T-cell engaging therapy either with chimeric antigen receptor (CAR) T-cells recognizing antigens expressed highly on MM cells or with bi-specific and tri-specific antibodies recognizing MM antigens and T-cells simultaneously.

Other novel drug classes include melphalan flufenamide (melflufen), a peptide-drug conjugate that delivers an alkylating agent directly into MM cells [83–86]. In a phase II trial of heavily pretreated patients, in combination with dexamethasone, melflufen had an ORR of 31% [87]. In an ongoing phase III trial of patients with RRMM, melflufen plus dexamethasone will be compared to pomalidomide and dexamethasone.

At the moment with only phase I and II data available for these emerging therapies, the temptation is to carry out cross-trial comparison in the penta-refractory setting in order to determine the optimum agent for an individual patient. As discussed above this approach is fraught with difficulties and can be misleading due to the composition of small biologically variable groups.

**Figure 3.**
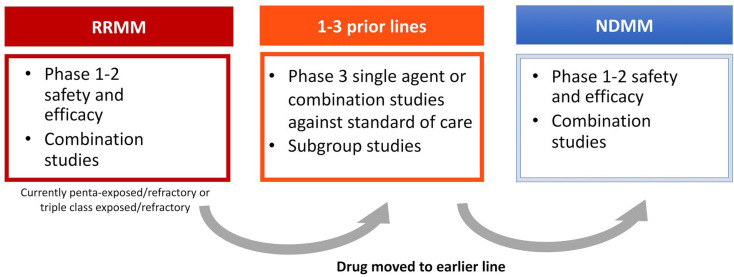
Traditional flow of drug development in MM

For pharmaceutical companies the challenge moving forward is how to develop a drug in such a crowded setting. There still remains an unmet need as patients continue to relapse, but do patients now need to be refractory to belantamab mafadotin and selinexor in order to receive these new agents? What happens in cases where it is not clinically appropriate that a patient receives all these drugs? Do therapies need to be given in a pre-determined order? For a phase III trial how is an appropriate comparator chosen? Is the “new RRMM” setting actually the most appropriate position to develop new agents? And finally, maybe most importantly, how does individual patient biology determine response to therapy? It will be critical to address these questions going forward for a disease where previously there was only one available therapy (Figure 3).

### The situation in patients not destined for transplantation

In older less-fit patients with MM who are not candidates for ASCT, drug development and off-label drug use has also occurred. Early studies explored combinations of low dose melphalan with a steroid followed by the addition of a third agent, such as thalidomide, bortezomib or lenalidomide. In this group of patients, it became obvious that combinations had the potential to be detrimental for frail patients, as the side effect profile reduced quality of life and decreased efficacy. The classic example of this was the combination of melphalan and prednisone with lenalidomide, where older patients did not achieve a survival benefit [88]. The FIRST trial established Rd as an alternative to a melphalan combination [89], leading eventually to the MAIA study which demonstrated that lenalidomide plus daratumumab (DRd) outperformed Rd, and importantly was not detrimental to quality of life in this frailer patient population [72]. The countrywide differences in the drug approval processes and marketing authorizations has led to quite dramatic differences in what is considered standard of care for this patient group. For example, in the US, off-label experience with dose-reduced VRD in older populations established the efficacy of this combination. This resulted in a clinical situation where VRD with daratumumab had largely become the standard for all groups, with performance status or frailty score being used to decide on the appropriate combination and dosing, whereas DRd, VMP and D-VMP are often used as standards in other countries.

### The impact of the European Union

For clarity we have focused predominantly on the evolution of therapies in the US, but of course, the European Union (EU), Canada, Asia-Pacific and South America exerted a significant impact on the use of therapies both on and off-label in the individual countries and in the US. Critical features impacting off-label use that differentiates these areas from the US are the different regulatory processes and the factors governing reimbursement in individual countries. For example, the EU regulatory authorities have tended to insist on evidence derived from randomized trials with PFS and OS as end points and have not had a fast track to licensing based on single arm studies in RRMM where response rate and PFS are end points. This has led to different time frames for approvals of new agents, different clinical trial designs to meet the evidence requirements of the regulatory agencies and the important role of country-specific data generated by many collaborative groups such as the IFM in France, MRC in the UK, Hovon in Holland, and the GMMG and DSSM in Germany. A key example of the country/regional difference is the use of VRD as a standard in the US, whereas in Europe, VTD and VMP were considered standards. Thus, when data emerged from large randomized studies based in Europe such as D-VTD and D-VMP they had little relevance in the US, and although FDA-approved were not widely utilized.

Perhaps the major factor driving the evolution of different preferred regimens in countries are the reimbursement procedures. With the complexity of drug costs and different healthcare systems, in addition to an assessment of safety and efficacy performed at the regulatory level, there is often an assessment of “value”, performed either at an insurance company level or a national level. For example, the European Medical Authority (EMA) may approve a license but the individual countries of the EU will then assess locally how the drug will be reimbursed within their country. The definition and methodologies behind the term “value” varies, as it takes in to account not only the expected benefit in terms of prolonged life for an individual patient, but the impact of this on the wider community (e.g., quality of life, productivity and contribution to the wider community, value in comparison to other health care advancements). It is therefore easy to see that with varying financial situations in the countries and different approval/reimbursement processes that large differences in both on and off-label prescribing can evolve. Ultimately though, the weight of the data and technological advances such as the recent introduction of anti-CD38 antibodies leads to a rebalancing of the system. This is in the process of happening with the global uptake of VRD combined with daratumumab as a global standard.

## Use of drugs that modulate novel targets that are labeled for use in another cancer

With increasing understanding of the genetic and molecular pathogenesis of MM, new potential therapeutic targets have emerged that may be amenable to inhibition by using drugs that have been developed for the same target in another cancer type. MM is characterized by recurrent chromosomal translocations involving the immunoglobulin heavy chain locus on chromosome 14, where genes are brought next to an immunoglobulin enhancer leading to dysregulated gene expression. For example, the t(4;14), present in 15% of MM [90, 91], leads to increased expression of the Wolf-Hirschhorn syndrome candidate gene (*WHSC1*, also known as *MMSET* and now *NSD2*) and the receptor tyrosine kinase fibroblast growth factor receptor 3 (*FGFR3*) gene [92, 93]. Inhibition of FGFR3 has been shown to induce plasma cell differentiation and apoptosis *in vitro* [94, 95].

Evaluating novel agents which may have activity in other cancers in MM where specific mutations are identified only in a small percentage of patients requires an “as yet to be determined standardized approach” to avoid further off-label prescriptions. Such an approach will probably involve an umbrella or basket trial design. An example is the ongoing NCI-MATCH study [96], which is “tumor pathogenic-type agnostic,” and uses molecularly targeted agents directed against the pathogenic mutation. As part of this trial, which did not include MM patients, AZD4547, an FGFR1-3 tyrosine kinase inhibitor was tested on 48 patients but did not meet its pre-specified efficacy endpoint for ORR.

Increasing knowledge derived from sequencing analysis of MM has provided more potential off-label targets. Mutational analysis of MM identified the central role played by mutations in the rat sarcoma (RAS)/mitogen activated protein kinase (MAPK) pathway suggesting it is a major target for therapeutic manipulation. Neuroblastoma-RAS (NRAS), Kirsten-RAS (KRAS) and BRAF are mutated in 25%, 25%, and 4–8% of cases respectively in NDMM [97, 98]. Mutations in these genes are one of the few genomic differences discovered to date between monoclonal gammopathy of undetermined significance (MGUS) and MM, suggesting an important role in the evolution from a precursor state to symptomatic disease. In a retrospective study of 40 patients with heavily pre-treated MM, nine had a complete response to off-label use of trametinib, an allosteric inhibitor of mitogen-activated protein kinase (MEK)1/2 [99]. Successful use of the BRAF inhibitor, vemurafenib, has also been seen, following the initial report in a patient with heavily pretreated MM and extramedullary disease [100]. Its use is currently being evaluated in a phase II study with preliminary results showing an 82% ORR [101].

The ongoing phase I/II Myeloma-Developing Regimens Using Genomics (MyDRUG) trial plans to leverage drugs approved for another indication [102]. MM patients with mutations in *CDKN2C*, *FGFR3*, *KRAS*, *NRAS*, *BRAFV600E*, *IDH2*, or the t(11;14) translocation are eligible. In combination with ixazomib, pomalidomide, and dexamethasone (IPd), based on their genomic targets enrolled patients will receive the drugs abemaciclib, enasidenib, cobimetinib, erdafitinib, or venetoclax. Patients without one of the targetable mutations described above will receive daratumumab in combination with IPd. This is not a registration trial for approval of these drugs for MM, but rather an example of testing therapies developed in other malignancies for use off-label.

## Can the story of thalidomide be recapitulated by repurposing other drugs for use in MM?

Given the poor prognosis of RRMM and the difficulty of approving novel drugs there is great interest in maximizing the use of a drug in more than one cancer indication as well as repurposing drugs already approved for another disease indication. The FDA ensures drugs are safe and effective, based on the Food, Drug, and Cosmetic Act of 1938 and the Kefauver-Harris Amendment, respectively [103]. Once approved for one indication, physicians are not prohibited from prescribing for indications or in doses not specified in the initial approval. The formal approval process for new drugs is long and costly. New drug approval is estimated to take 10–20 years [104] and cost up to $2 billion [105]. The large majority of these drugs fail during clinical development: between 2003 and 2011 an estimated 6.7% of drugs tested in a phase I trial for an oncologic indication made it to FDA approval [106]. At the same time, the cost of new cancer therapies is rising, contributing to increasing financial costs for both individual patients and to healthcare systems [107, 108].

Considering the cost and difficulty of developing novel therapies, one strategy has been the repurposing of drugs already available for a different disease. Through programs such as the ReDO project [109] there is ongoing work to repurpose existing low-cost drugs. The anti-helmenthinc mebendazole, for example, has been shown *in vitro* and in mouse models to inhibit tumorogenesis in lung cancer [110, 111], adrenal tumors [112], melanoma [113], glioblastoma multiforme [114], and breast cancer [115]. In MM, mebendazole delayed tumor growth in mice, possibly through the USP5/c-Maf pathway [116]. Nocodazole, another anti-helmenthic, inhibited tumor growth via cell cycle arrest and microtubule dysfunction, associated with increased Bim and myeloid cell leukemia-1 (MCL-1) expression and c-Jun NH2-terminal kinase (JNK)-mediated B cell lymphoma protein-2 (BCL-2) phosphorylation [117]. Despite these promising *in vitro* results it appears the introduction of these agents into the clinic is unlikely, but the examples demonstrate the possibilities of such an approach.

Another example is the antibiotic clarithromycin, which shows high response rates when combined with Rd [118], and had a higher response rate than Rd alone when compared retrospectively [119]. A phase II trial evaluated clarithromycin combined with pomalidomide and dexamethasone and showed a 60% ORR [120]. Proposed mechanisms for clarithromycin’s effect include increasing the concentration of dexamethasone through cytochrome P450 3A4 (CYP3A4) inhibition, downregulation of T-reg cell response via decreased interleukin (IL)-6 and increased IL-10 and interferon (IFN)-y levels [121, 122], and through attenuation of autophagy in MM cells [123]. Despite the enthusiasm concerning the initial results the combination has not been widely taken up because of uncertainty about the mechanism or its clinical relevance.

The combination of the protease inhibitor ritonavir with metformin has shown antitumor activity in MM both *in vitro* and *in vivo* with xenograft mouse models, through inhibition of glucose transporter 4 (GLUT4) and mitochondrial oxidative metabolism, leading to suppression of signaling through the protein kinase B (AKT)/mammalian target of rapamycin complex 1 (mTORC1) pathway known to regulate MCL-1 [124]. Ritonavir has also been shown to sensitize MM cells to the effect of bortezomib [125]. Early trial data showed response rates to nelfinavir, another protease inhibitor, in patients who were previously exposed and resistant to bortezomib [126, 127]. This approach has not been widely taken up because of the subsequent development of agents targeting BCMA, however the clinical experience with the combination was that it could be useful and was not toxic, making these agents potential alternatives if recently approved agents were not available.

A further example is venetoclax, a BCL-2 inhibitor that is approved for use in chronic lymphocytic leukemia (CLL) and acute myelogenous leukemia (AML). Early clinical trials in RRMM, as well as case reports in plasma cell leukemia and amyloid disease show it is particularly effective in patients with a t(11;14) [128–130]. Its development in MM has been checkered as the early studies investigated all MM patients, not just t(11;14) cases, and although it was found to be more efficacious in combination with Vd compared to Vd alone, it was also noted to have increased toxicity leading to a pause in development [131]. Studies have now re-commenced in the group of patients where retrospective case reports had shown unprecedented activity—those with t(11;14)—and it is hoped the drug will eventually gain formal approval in this space.

## Conclusion

In the earlier era of alkylating agents and corticosteroids, off-label drug use filled a void in available therapies and was critical to the improved outcomes seen in MM today. With the advances in the number of active MM therapies, the clinical trial and regulatory approval framework has continually evolved to accommodate the safe and effective investigation and approval of new therapies. Moving forward we firmly believe that patients wherever possible should be treated within the context of clinical trials, that off-label use should be kept to a minimum, and safety and efficacy data reported. We do however recognize that off-label use continues in MM therapy. Currently, a frequent and notable off-label use is during development of novel therapies where use of a therapy becomes widespread following the release of promising clinical trial results prior to regulatory approval. With further understanding of the molecular and cellular mechanisms of MM and cancer pathogenesis generally there has been a move toward targeted therapies being used across multiple cancer types. This has led to a further off-label area for “niche therapies” for small well-defined patient populations where large clinical trials with sufficient power may be difficult to perform. Finally, we have seen that drugs initially used for non-neoplastic indications have activity in MM, leading to the possibility of cheap and safe drugs already in production becoming effective components of combination therapy. Cautionary use of off-label prescribing has provided and will continue to provide a vehicle to enhance the treatment of MM patients with the introduction of effective treatment regimens in a disease setting where prognosis is poor and there remains an unmet medical need.

## Abbreviations

ASCT: autologous stem cell transplantation
BCMA: B-cell maturation antigen
DRd: daratumumab-Revlimid-dexamethasone
D-VMP: daratumumab-Velcade-melphalan-prednisone
D-VTD: daratumumab-Velcade-thalidomide-dexamethasone
EU: European Union
FDA: Food and Drug Administration
FGFR3: fibroblast growth factor receptor 3
HDM: high-dose melphalan
IMiD: immunomodulatory drug
MM: multiple myeloma
MP: melphalan-prednisone
NDMM: newly diagnosed multiple myeloma
ORR: overall response rate
OS: overall survival
PFS: progression free survival
PI: proteasome inhibitor
Rd: Revlimid-dexamethasone
RRMM: relapsed/refractory multiple myeloma
VAD: vincristine-doxorubicin-dexamethasone
Vd: Velcade-dexamethasone
VMP: Velcade-melphalan-prednisone
VRD: Velcade-Revlimid-dexamethasone
VTD-PACE: Velcade-thalidomide-dexamethasone-cisplatin-doxorubicin-cyclophosphamide-etoposide

## Declarations

### Copyright

© The Author(s) 2021.
